# The prevalence of comorbidities in Danish patients with obesity – A Danish register‐based study based on data from 2002 to 2018

**DOI:** 10.1111/cob.12542

**Published:** 2022-06-29

**Authors:** Mikkel H. Pedersen, Mette Bøgelund, Carsten Dirksen, Pierre Johansen, Nils B. Jørgensen, Sten Madsbad, Ulrik H. Pantin

**Affiliations:** ^1^ Incentive Holte Denmark; ^2^ Department of Endocrinology Hvidovre Hospital Hvidovre Denmark; ^3^ Novo Nordisk North West Europe Pharmaceuticals A/S Copenhagen Denmark

**Keywords:** burden of illness, comorbidities, obesity

## Abstract

We used the Danish National Health Registers to conduct a study on the prevalence of obesity‐related comorbidities in Danish citizens who have been diagnosed with obesity at a Danish hospital. This was a retrospective observational study with a population comprising all Danish citizens (≥18 years) who have been registered with a specific obesity class diagnosis in the Danish National Patient Register between 2002 and 2018. A total of 86 980 persons with hospital‐diagnosed obesity were included in the study population. To investigate how the risk of having comorbidities varies with the degree of obesity, we applied adjusted logistic regression to estimate the odds ratio of having one of the following predefined comorbidities for people with a BMI in obesity classes II and III compared with people with a BMI in obesity class I: type 2 diabetes, ischaemic heart disease, non‐alcoholic steatohepatitis and non‐alcoholic fatty liver disease, hip and knee osteoarthritis, obstructive sleep apnoea and asthma. Comorbidities were defined from ICD‐10 diagnosis codes and prescription medication utilization. The odds ratio for obstructive sleep apnoea (OR 1.86 and OR 3.0), type 2 diabetes (OR 1.68 and OR 2.26), hip and knee osteoarthritis (OR 1.29 and OR 1.54) and asthma (OR1.13 and OR 1.25) increased significantly with obesity class (obesity class II relative to I and III relative to I, respectively). The odds ratio of having had at least one comorbidity was estimated to be 1.52 for people with a BMI in obesity class II and 2.10 for people with a BMI in obesity class III compared with people in obesity class I. The risk of obstructive sleep apnoea, type 2 diabetes, hip and knee osteoarthritis, and asthma increased significantly with increasing BMI, highlighting the importance of preventing further weight gain even in individuals who are already living with obesity.

**What is already known about this subject:**

- The prevalence of obesity is increasing.
- Obesity results in an increased risk of severe health consequences.
- Obesity is associated with a high number of comorbidities.

**What this study adds:**

- A thorough evaluation of the risk of having obesity‐related comorbidities in relation to BMI classes I, II and III.
- The risk of obstructive sleep apnoea, type 2 diabetes, hip and knee osteoarthritis, and asthma increases with increased BMI.

## 1 INTRODUCTION

The prevalence of obesity (defined as a body mass index or BMI, above 30 kg/m^2^) in the adult population has increased substantially over the past decades.^1^ The World Health Organization (WHO) estimates that obesity affects 23% of the population in the European Region and the UK combined.^2^ From 1975 to 2014, an increase in the number of both men and women with a BMI in obesity class I (30 to <34.9 kg/m^2^) and obesity class II (35 to <39.9 kg/m^2^) was reported amongst Europeans.^3^ In several other parts of the world, the number of people with a BMI in obesity class III (>40 kg/m^2^) is rapidly increasing.^3^, ^4^ It is projected that by 2025, obesity will have increased considerably in 44 of the 53 WHO Member States in the European Region and the UK.^2^

In Denmark, the 2017 self‐reported obesity rate was estimated at 17% of the adult population (corresponding to 700 000 persons),^5^ and the WHO forecasts that 27% of Danish men and 26% of Danish women will have a BMI above 30 in 2030.^6^

Obesity results in an increased risk of severe health consequences and mortality and has been associated with more than 200 comorbidities and substantial health care costs.^7^, ^8^ Apovian (2016)^9^ reported that people with obesity are at increased risk of dyslipidaemia, type 2 diabetes (T2D), hypertension, coronary heart disease, stroke, gallbladder disease, respiratory problems, sleep apnoea, osteoarthritis, major depressive disorder and some cancers, based on a review of the literature. Moreover, Apovian concluded that adults with a BMI in obesity class III were at particularly high risk of T2D, hypertension, hyperlipidaemia, asthma and arthritis.^9^ Andreyeva et al. (2004)^10^ concluded that the risks of comorbidities and severity of conditions associated with obesity increase rapidly with increasing BMI. Kjellberg et al. (2017)^11^ investigated the odds ratio (OR) for comorbidities per BMI point above 30 in a Danish setting using self‐reported height and weight from the 2013 Danish National Health Profile Survey. They found an association between increasing BMI above 30 and risk for comorbidities.

Thus, it is well‐known that obesity is associated with a high number of comorbidities.^7^, ^10^, ^11^ However, there is less evidence on the disease burden amongst patients with hospital‐diagnosed and categorized obesity. In this study, we investigated the burden of disease in Danish people with obesity by identifying which comorbidities were the most prevalent, and to what extent the risk of developing obesity‐related comorbidities was associated with increasing BMI, in order to evaluate the need for early disease‐specific interventions, amongst people with hospital diagnosed obesity. To our knowledge, this is the first large‐scale study identifying the prevalence of comorbidities amongst individuals with obesity in Denmark using BMI registrations from the Danish National Health Registers.

## 2 MATERIALS AND METHODS

### 2.1 Study population

Data on all adults (≥18 years) admitted to the hospital (outpatient or hospital admission) and registered with a diagnosis code indicating a specific BMI classification (identified by the ICD‐10 codes E660B‐E660H) as a primary or secondary diagnosis in the years 2002 to 2018 were identified in the Danish National Patient Register (DNPR). Women who were registered with obesity only in relation to pregnancy were excluded from the study population (ICD‐10 codes O and Z3, including sublevels).

The study population was categorized according to the WHO's classification of obesity: obesity class I (BMI 30 to 34.9 kg/m^2^, ICD‐10 code E660B), obesity class II (BMI 35 to 39.9 kg/m^2^, ICD‐10 code E660C‐D) and obesity class III (BMI ≥ 40 kg/m^2^, ICD‐10 code E660E‐H). For this study, all people were assigned to one of the three obesity classes based on their first registration indicating an obesity class.

### 2.2 Outcome variables

Following the guidelines from The Danish Health Data Authority (SDS),^12^ we identified a total of 50 comorbidities, including cancers, cardiovascular diseases and non‐cardiovascular diseases. A patient was defined as having a comorbidity if he or she was registered with at least one hospital contact (inpatient hospitalization or outpatient contact) for which the primary or secondary diagnosis was listed with the relevant ICD‐10 code or had filled a prescription for disease‐specific prescription medication. Likewise, a patient was defined as having a comorbidity related to mental health disorders if he or she was registered with at least one hospital contact at a psychiatric hospital or a psychiatric ward in a general hospital. Mental health disorders were defined per the Danish Psychiatric Central Register.

For the calculation of the OR of having comorbidities amongst obesity classes, outcome variables included the following six predefined frequent obesity‐related comorbidities: obstructive sleep apnoea (OSA), T2D, non‐alcoholic steatohepatitis/non‐alcoholic fatty liver disease (NASH/NAFLD), hip and knee osteoarthritis, asthma and ischaemic heart disease (IHD). These were purposefully selected from a comprehensive list of obesity‐related disorders to represent common obesity‐related complications.^7^, ^8^ In addition to this, comorbidities were selected to be comorbidities that would at some point most likely require disease‐specific prescription medication or hospitalization and therefore are identifiable in the Danish registers.

### 2.3 Data source

In the Danish Civil Registration System (CRS), each Danish citizen is assigned a unique personal identification number.^13^ The CRS enables an identity‐secure linkage of information amongst the Danish national registers. Information on all somatic hospitalizations in Denmark has been recorded in the Danish National Patient Registry (DNPR) since 1977,^14^ and all somatic outpatient activities and emergency room contacts, including diagnoses and performed procedures, since 1995.^14^ At each record, information on the primary diagnosis related to the hospital contact and up to 19 secondary diagnoses is registered. Mental health disorders have also been recorded since 1995 in a separate register, the Danish Psychiatric Central Register.

### 2.4 Statistical analyses

The study included data on patients' comorbidities from 5 years before the date of the first diagnosis of obesity (i.e. index date) to 15 years after the index date, patient death, patient emigration or end of follow‐up (31 December 2018), whichever occurred first. The median follow‐up was 5.19 years. The rationale for following study participants from 5 years prior to the index date was that obesity is a chronic condition. In this regard, it is considered likely that participants were obese in the 5 years prior to the recording of the obesity diagnosis.

Initial descriptive analyses were used to characterize the study population within each obesity class. Any registration of comorbidities in the study period was used as the basis for computing the prevalence of comorbidities in the three obesity classes. Within each obesity class, patients were grouped according to the number of comorbidities diagnosed in the period from 5 years prior to the person's first obesity‐related hospital contacts to 5 years after that first contact (0 comorbidities, 1–3 comorbidities, 4–6 comorbidities, 7–10 comorbidities and 10 or more comorbidities). Adjusting for age and gender, logistic regression analysis was applied to estimate the OR of having had a hospital contact with primary or secondary diagnosis of each of the six predefined obesity‐related comorbidities for people with a BMI in obesity classes II and III compared with people with a BMI in obesity class I. People with a BMI in obesity class I were selected as the reference group in the analysis, to minimize sample selection bias.

Sensitivity analyses were performed to explore robustness and for comparison purposes and estimated the OR of the predefined comorbidities according to obesity class and stratified by gender, age and educational attainment.

Data were analysed using the framework for access to microdata at Statistics Denmark. Data were accessed and analysed on the research computers at Statistics Denmark via a secure connection. All analyses were performed in SAS 9.4.

### 2.5 Ethical considerations

The study was register based and complied with the regulations set up by the Danish Data Protection Agency (J. no. 2014‐54‐0664). No ethical approval was needed.

## 3 RESULTS

### 3.1 Study population

Patient characteristics of the study population are presented in Table 1. A total of 86 980 persons with hospital‐diagnosed obesity were included in the study population. There were 42 372 persons identified with a BMI in obesity class I (80% female, mean age at obesity diagnosis 52.7 years); 28 014 persons with a BMI in obesity class II (78% female, mean age at diagnosis 49.9 years) and 16 594 with a BMI in obesity class III (73% female, mean age at diagnosis 47.6 years).

**TABLE 1:** Patient characteristics by obesity class

	Obesity class I, *n* (%)	Obesity class II, *n* (%)	Obesity class III, *n* (%)
Total	42 372	28 014	16 594
Gender			
Male	8358 (20%)	6278 (22%)	4549 (27%)
Female	34 014 (80%)	21 736 (78%)	12 045 (73%)
Age at diagnosis			
Mean age (SD)	52.69 (15.48)	49.9 (14.93)	47.59 (14.63)
<40 years	8898 (21%)	7435 (27%)	5237 (32%)
45–65 years	23 414 (55%)	15 765 (56%)	9169 (55%)
Above 65 years	10 060 (24%)	4814 (17%)	2188 (13%)
Educational level			
Primary or no education	6150 (37%)	10 326 (37%)	14 468 (34%)
High school	750 (5%)	1024 (4%)	1466 (3%)
Upper secondary	6193 (37%)	10 805 (39%)	16 536 (39%)
Bachelor's or equivalent	2824 (17%)	4601 (16%)	7766 (18%)
Master's or higher	345 (2%)	634 (2%)	1248 (3%)
Education unknown	332 (2%)	624 (2%)	888 (2%)
First hospital registration of obesity			
Mean index year (SD)	2012 (3.74)	2012 (3.98)	2014 (2.51)

*Note*: The table presents sociodemographic characteristics for persons with hospital‐diagnosed obesity included in the study population.

### 3.2 Risk of comorbidities by type of comorbidity

OR estimates associated with the six preselected comorbidities are presented in Table 2 and in a forest plot in Figure 2. They illustrate the risk of having the specific type of comorbidity for people with a BMI in obesity classes II and III relative to people with a BMI in obesity class I. The age‐ and gender‐adjusted OR of having been registered with a comorbidity increased significantly with each obesity class for four of the six predefined comorbidities; obstructive sleep apnoea (OR obesity class II: 1.86 (1.76–1.97); OR obesity class III: 3.00 (2.82–3.18)), T2D (OR obesity class II: 1.68 (1.62–1.74); OR obesity class III: 2.26 (2.16–2.36)), hip and knee osteoarthritis (OR obesity class II: 1.29 (1.23–1.34); OR obesity class III: 1.54 (1.46–1.62)), and asthma (OR obesity class II: 1.13 (1.07–1.18); OR obesity class III: 1.25 (1.19–1.32)).

**TABLE 2:** Odds ratio estimates the risk of having comorbidities by type of comorbidity

	Adjusted OR			Obesity class I	Obesity class II		Obesity class III	
	Obesity class II	Obesity class III	Obesity class III*	Prevalence	Prevalence	Adjusted prevalence	Prevalence	Adjusted prevalence
Obstructive sleep apnoea	1.86 (1.76–1.97)	3.00 (2.82–3.18)	1.61 (1.52–1.71)	6.6%	11.7%	11.6%	17.1%	17.5%
T2D	1.68 (1.62–1.74)	2.26 (2.16–2.36)	1.34 (1.28–1.41)	22.4%	30.7%	32.7%	29.9%	39.4%
NASH/NAFLD	1.12 (0.96–1.31)	1.15 (0.95–1.39)	1.02 (0.84–1.24)	0.9%	1.0%	1.0%	1.0%	1.0%
Hip and knee osteoarthritis	1.29 (1.23–1.34)	1.54 (1.46–1.62)	1.20 (1.14–1.27)	20.4%	22.1%	24.8%	18.8%	28.3%
Asthma	1.13 (1.07–1.18)	1.25 (1.19–1.32)	1.11 (1.05–1.17)	17.4%	20.3%	19.2%	19.7%	20.9%
Ischaemic heart disease	1.03 (0.97–1.09)	0.94 (0.87–1.02)	0.92 (0.84–1.00)	8.9%	7.9%	9.1%	5.3%	8.4%
≥1 of the defined comorbidities	1.52 (1.47–1.57)	2.10 (2.02–2.19)	1.39 (1.33–1.45)	54.0%	62.2%	64.0%	61.7%	71.2%
None of the defined comorbidities	0.66 (0.64–0.68)	0.48 (0.46–0.50)	0.72 (0.69–0.75)	46.0%	37.8%	36.0%	38.3%	28.9%

*Note*: The table presents the odds ratio (95% confidence interval) and unadjusted and age‐ and gender‐adjusted prevalence of having the predefined comorbidities. Age‐ and gender‐adjusted OR for obesity class II and obesity class III relative to obesity class I. The OR for obesity class III* is relative to obesity class II. Adjusted prevalence was calculated by (OR/[1‐risk obesity class1 + risk obesity class1*OR])*risk obesity class I.

People with BMI in obesity class II had 52% increased odds of having at least one of the six comorbidities compared with people with a BMI in obesity class I. For people with a BMI in obesity class III, the relative risk of having had at least one comorbidity was more than twice that of the reference group (BMI class I). People with a BMI in obesity class III had a threefold risk of OSA compared with people with a BMI in obesity class I; OSA was identified as the comorbidity associated with the greatest OR estimate in both BMI obesity class II and class III.

The unadjusted prevalence of OSA was 6.6%, 11.7% and 17.1% in obesity classes I, II and III, respectively. The unadjusted prevalence of T2D was 22.4%, 30.7% and 29.9% in obesity classes I, II and III, respectively, making T2D the most common comorbidity in all three obesity classes.

### 3.3 Number of patients with any comorbidity by obesity class

**FIGURE 1:**
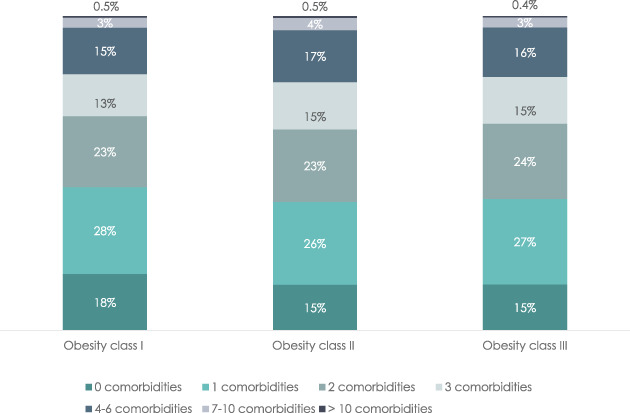
Number of any comorbidity by obesity class. *Note*: Percentage of the study population with 0, 1–3, 4–6, 7–10 and >10 comorbidities for each obesity class

Figure 1 shows the distribution of patients with a BMI in each of the three obesity classes by the number of registered comorbidities out of the 50 comorbidities, including cancers, cardiovascular diseases and non‐cardiovascular diseases. Of the total population, 85.2% had one or more comorbidities, with just over 25% in each obesity class having just one comorbidity and 23%–24% having two comorbidities. Obesity classes also did not differ in the percentage of people with 0, 3, 4–6, 7–10 and >10 comorbidities and only 0.5% in each obesity class had >10 comorbidities.

**FIGURE 2:**
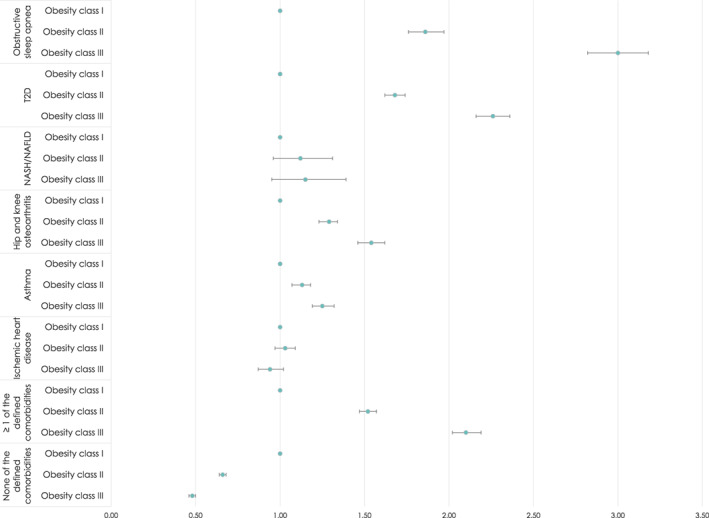
Forest plot of odds ratio estimates the risk of having comorbidities by type of comorbidity. *Note*: Age‐ and gender‐adjusted OR for obesity class II and obesity class III relative to obesity class I.

### 3.4 Sensitivity analyses

Sensitivity analyses estimating the OR of having had obesity‐related comorbidities across gender, age and educational attainment are presented in Table 3. In all specified subgroups, the OR of having been registered with OSA or T2D as a comorbid condition to obesity as well as having been registered with at least one of the predefined comorbidities increased significantly with each obesity class. In line with the results from the base case analysis, the estimated OR of having been registered with asthma or hip and knee osteoarthritis as a comorbid condition to obesity increased with each obesity class but was not statistically significant in the smaller subgroups of males, persons aged 55 and above, and amongst persons with a master's degree or higher education.

**TABLE 3:** Sensitivity analyses: Odds ratio estimates of the risk of having comorbidities by sub‐population and type of comorbidity

Subgroup	Obstructive sleep apnoea	T2D	NASH/NAFLD	Hip and knee osteoarthritis	Asthma	Ischaemic heart disease	≥1 of the defined comorbidities	None of the defined comorbidities
Males								
Obesity class II	1.92***	1.66***	0.86	1.07	1.03	0.92*	1.51***	0.66***
Obesity class III	2.74***	2.09***	0.84	1.22***	1.05	0.72***	2.05***	0.49***
Obesity class III^a^	1.42***	1.26***	0.98	1.14**	1.01	0.78***	1.36***	0.74***
Females								
Obesity class II	1.80***	1.68***	1.21**	1.35***	1.15***	1.07**	1.52***	0.66***
Obesity class III	3.20***	2.32***	1.24*	1.65***	1.30***	1.16**	2.12***	0.47***
Obesity class III^a^	1.77***	1.38***	1.03	1.22***	1.13***	1.07	1.40***	0.72***
Age < 35								
Obesity class II	2.05***	1.49***	1.45**	1.24*	1.11**	0.88	1.31***	0.77***
Obesity class III	4.16***	1.97***	1.21	2.06***	1.30***	0.73	1.83***	0.55***
Obesity class III^a^	2.03***	1.32***	0.83	1.66***	1.18***	0.83	1.40***	0.72***
Age 35–55								
Obesity class II	1.77***	1.75***	1.00	1.47***	1.17***	0.94	1.62***	0.62***
Obesity class III	2.79***	2.50***	1.02	1.97***	1.26***	0.83**	2.36***	0.43***
Obesity class III^a^	1.57***	1.43***	1.02	1.34***	1.08**	0.89	1.45***	0.69***
Age > 55								
Obesity class II	1.81***	1.61***	1.09	1.15***	1.05	1.05	1.48***	0.67***
Obesity class III	2.86***	2.08***	1.25	1.24***	1.19***	0.98	1.92***	0.52***
Obesity class III^a^	1.58***	1.29***	1.15	1.07*	1.13**	0.93	1.29***	0.78***
Primary education or missing								
Obesity class II	1.95***	1.63***	0.98	1.23***	1.10***	1.06	1.49***	0.67***
Obesity class III	2.95***	2.07***	0.97	1.44***	1.23***	0.96	1.98***	0.51***
Obesity class III^a^	1.51***	1.27***	0.99	1.17***	1.12**	0.91	1.33***	0.75***
High school, secondary education, bachelor's or equivalent								
Obesity class II	1.80***	1.68***	1.13	1.29***	1.12***	0.97	1.48***	0.68***
Obesity class III	3.08***	2.27***	1.11	1.60***	1.25***	0.89	2.15***	0.47***
Obesity class III^a^	1.71***	1.35***	0.98	1.24***	1.13***	0.92	1.45***	0.69***
Master's degree or higher education								
Obesity class II	1.91***	1.71***	1.51**	1.47***	1.28***	0.92	1.65***	0.61***
Obesity class III	3.02***	2.48***	1.75**	1.66***	1.27***	0.81	2.10***	0.48***
Obesity class III^a^	1.58***	1.45***	1.16	1.13	1.00	0.87	1.28***	0.78***
Index date before 2013								
Obesity class II	1.84***	1.81***	1.02	1.33***	1.14***	1.09**	1.55***	0.65***
Obesity class III	2.53***	2.38***	1.22	1.49***	1.18***	0.97	1.89***	0.53***
Obesity class III^a^	1.38***	1.32***	1.20	1.12**	1.04	0.89	1.22***	0.82***
Index date after 2013								
Obesity class II	1.98***	1.66***	1.51***	1.35***	1.17***	1.00	1.52***	0.66***
Obesity class III	3.83***	2.44***	1.53***	1.68***	1.40***	0.95	2.32***	0.43***
Obesity class III^a^	1.93***	1.47***	1.01	1.25***	1.20***	0.95	1.53***	0.65***

*Note*: The table presents the odds ratio of the predefined comorbidities according to obesity class and stratified by gender, age and educational attainment. Estimates were significant at *10%, **5%, ***1%.

^a^ Odds ratio for obesity class III relative to obesity class II.

The OR for having been registered with either NASH/NAFLD or IHD was mostly estimated to be not statistically different from 1, although the OR for having been registered with IHD was found to decrease statistically significantly with obesity class amongst men and increase statistically significantly with obesity class amongst women.

## 4 DISCUSSION

In this study, we have provided evidence on the long‐term burden of disease in Danish people with BMI > 30 by identifying which comorbidities were the most prevalent, and to what extent the risk of developing obesity‐related comorbidities was associated with increasing BMI. We estimated the risk of having had at least one of the six prespecified comorbidities to be significantly higher for people with a BMI in obesity classes II and III compared with people in obesity class I. The sensitivity analysis showed that the estimates of the OR of having specific comorbidities were similar across subgroups based on gender, age and educational attainment. Even so, IHD decreased with increased obesity class in men, whereas it increased in women. It is outside the scope of this study to account for this gender difference, but one possible explanation is that this difference is linked to the difference in women's and men's health‐related and help‐seeking behaviour.^15^

The study included all adults (≥18 years) who had been registered with a specific BMI class diagnosis code in the obese range at a Danish hospital. Studying a similar population, Kjellberg et al.^11^ found that an increase in BMI was associated with a significant increased risk of diseases of the blood and blood‐forming organs, endocrine, nutritional and metabolic diseases, diseases of the nervous system and diseases of the circulatory system. Our study extends these findings by studying the impact of BMI on a set of specific frequent comorbidities as well as on the burden of disease in persons with obesity.

Our findings indicate that increased BMI amongst people with obesity is to a large extent associated with the development of serious chronic comorbidities, which is both costly for society and discomforting for patients, such as OSA, T2D, hip and knee osteoarthritis, and asthma. These findings are in line with what has previously been reported by Apovian and others, who describe an increased risk of morbidity from, amongst other conditions, T2D, osteoarthritis, asthma and sleep apnoea in people with obesity.^8^, ^9^ The increased risk of developing chronic conditions such as T2D, asthma and osteoarthritis with increased BMI is likely linked to ectopic fat accumulation, endocrine dysfunction including insulin resistance, and low‐grade inflammation^16^ as well as the excess load placed on joints.

Moreover, our findings suggest a high burden of comorbid disease, i.e. number of comorbid diagnoses, in patients with hospital‐diagnosed obesity that to a lesser extent dependent on obesity class. This finding is somewhat contradictory to our finding of an association between increased obesity class and greater risk of obesity‐specific comorbid conditions, and it also contradicts what is usually reported in obesity research, where increased BMI is linked to increased risk of comorbidities. However, this finding is likely to be explained by the inclusion of people with hospital‐diagnosed obesity. People are often diagnosed with obesity in relation to comorbidities—therefore, the cases in this study, regardless of obesity class, might include those who to a greater extent suffer from comorbid conditions.

Hence, our results suggest that although the general burden of disease is high in patients with obesity, specific comorbidities are much more strongly associated with increased BMI. This highlights the importance of further research into the causal relationship between the degree of obesity and the risk of developing specific comorbidities in order for decision makers and health authorities to initiate effective and targeted intervention in the early stages of obesity to limit the risk of future comorbidities and the costs associated with the treatment of these.

### 4.1 Limitations

The study was limited to the information available in the Danish registers. The Danish registers contain obesity information only on people who received a BMI‐specific diagnosis of obesity at a hospital. Approximately 780 000 people live with obesity in Denmark, thereby, our sample constitutes 11% of all people with obesity in Denmark.^17^ As such, the study omitted people with an unspecified obesity diagnosis (ICD‐10 codes DE0660 and DE669), which limited the sample size. This inclusion restriction was made to increase the likelihood that the registration of a specific BMI class was based on an actual measurement of weight and height. The use of BMI as a measure of obesity classification has some limitations because it does not reflect the relation between body fat and lean mass and might not reflect bodily differences as a result of e.g. aging or sex.^18^ However, it is standard practice to measure obesity with BMI in many studies. Additionally, the registration of a BMI‐specific diagnosis in the Danish registers requires an assessment from health professionals; therefore, we believe that the impact of the limitations surrounding BMI has minimal effect on the results in the present study.

It is likely that persons who have received a hospital diagnosis of obesity are those with more severe comorbidities, which could potentially impact the estimates of the prevalence of comorbidities. At the same time, this also implies that we were only able to identify individuals as having had the specific comorbidity if the comorbidity had been registered during a hospital contact. To minimize the impact of this limitation, the comorbidities of primary interest were selected to be comorbidities that would at some point most likely require disease‐specific prescription medication or hospitalization and therefore are identifiable in the Danish registers.

In the study, we compared the OR of having been registered with a specific comorbidity for persons with a BMI in obesity classes II and III relative to persons with a BMI in obesity class I. Persons were classified into one of the three obesity classes based on their first registration with a diagnosis code indicating the BMI of the person. Thereby, the present study did not account for possible changes in a person's BMI over time. In case the BMI of a person increases over time, this would cause us to underestimate the true impact of BMI on the risk of having comorbidities.

In addition to this, the study was based on cross‐sectional data, and for that reason is unable to provide a causal relationship between BMI and the risk of being registered with comorbid conditions. Further research on the association and causation of obesity and comorbidities is needed.

### 4.2 Strengths

The use of Danish registers for retrieval of real‐world data allowed us to include all Danish citizens aged 18 years and above with a hospital diagnosis of obesity, thereby ensuring the robustness of the results.

In addition to this, the inclusion of data on the consumption of disease‐specific medication enables us to reliably identify comorbidities that would not have been captured in the hospitalization data.

## CONFLICT OF INTEREST

This work was supported by Novo Nordisk North West Europe, Denmark. Pierre Johansen and Ulrik Panton are employees of Novo Nordisk North West Europe Pharmaceuticals A/S, Copenhagen and shareholders of Novo Nordisk stocks. Mikkel H. Pedersen and Mette Bøgelund are employees of Incentive, which is a paid vendor of Novo Nordisk North West Europe Pharmaceuticals. Carsten Dirksen and Nils Jørgensen both sit on a Novo Nordisk advisory board and further have received lecture fees. Carsten Dirksen has received support for attending meeting/congress. Nils Jørgensen is a member of the board of the Danish Association for the Study of Obesity and is a shareholder of Novo Nordisk and Eli Lilly stocks. Sten Madsbad has nothing to declare.

## Supporting information


Appendix S1
