# Supplementary material for: The prevalence of comorbidities in Danish patients with obesity – A Danish register‐based study based on data from 2002 to 2018
Source: Clin Obes. 2022 Jun 29;12(5):e12542. doi: 10.1111/cob.12542 (PMC9539517; doi:10.1111/cob.12542)
Supplement: Supplementary file 1 — Appendix S1 [file COB-12-e12542-s001.pdf]

The prevalence of comorbidities in Danish patients with obesity – a Danish register-based study based on data from 2002 to 2018

Pedersen Mikkel H.[a], Bøgelund Mette[a], Dirksen Carsten[b], Johansen Pierre[c], Jørgensen Nils B.[b], Madsbad Sten[b], Panton Ulrik H.[c]

[a]Incentive, Holte Stationsvej 14, 1, 2840 Holte, Denmark.
[b]Department of Endocrinology, Hvidovre Hospital, Kettegaard Alle 30, 2650 Hvidovre, Denmark.
[c]Novo Nordisk North West Europe Pharmaceuticals A/S, Crowne Plaza Copenhagen Towers, Ørestads Blvd. 108, 2300 Copenhagen, Denmark.

Corresponding author:

Mikkel H. Pedersen,
T. +45 6017 7416
E. mp@incentive.dk
A: Holte Stationsvej 14, 1, 2840 Holte, Denmark

Appendix 1: Definition of comorbidities

Table A1: Definition of CV-related comorbidities

**Comorbidity**		**ICD-10 diagnosis code**		**ATC code for relevant prescription medicines**
**Myocardial infarction**	ICD:	I21-23		
**Ischemic heart disease (revascularisation)**	ICD:	I24-25		
**Unstable angina**	ICD:	I20.0		
**Angina pectoris**	ICD:	I20.1, I20.8, I20.9		
**Atrial fibrillation**	ICD:	I48	ATC:	C01B
**Heart failure**	ICD:	I50		
**Stroke**	ICD:	I60-I66		
Haemorrhage	ICD:	I60-I62		
Ischemic	ICD:	I63-I64		
Transient cerebral ischaemic attacks	ICD:	G45		
**Peripheral artery disease**	ICD:	I70-I71		
**Heart failure with preserved ejection fraction, HFpEF**	ICD:	I503		
**Heart failure**	ICD:	I50		
**Hypertension**			ATC:	C02

Table A2: Definition of other comorbidities

Comorbidity	ICD-10 diagnosis code	ATC code for relevant prescription medicines
**Gout**	ICD: M10	
**Asthma**		**See appendix 2*
**Atherosclerosis**		ATC: B01
**Chronic kidney disease**	ICD: N18	
Stage I	ICD: N18.1	
Stage II	ICD: N18.2	
Stage III	ICD: N18.3	
Stage IV	ICD: N18.4	
Stage V	ICD: N18.5	
**Depression**	ICD: F32.9, F33.9	ATC: N05-N06
**Dementia**		**See appendix 2*
**Diabetes Type I**		**See appendix 2*
**Gallstones**	ICD: K563	
**Gastroesophageal reflux**	ICD: K21	
**Hidradenitis suppurativa**	ICD: L73.2	
**Idiopathic intracranial hypertension**	ICD: G93.2	
**Knee/hip osteoarthritis**	ICD: M16, M17	
**Polycystic ovary syndrome, PCOS**	ICD: E282	
**Female infertility**	ICD: N97	
**Osteoarthritis**		**See appendix 2*
**Obstructive sleep apnoea**	ICD: G473	
**Psoriasis**	ICD: L40	ATC: D05
**Pancreatitis**	ICD: K85	
**Disorder of urinary system**	ICD: N39	
**Non-alcoholic steatohepatitis, NASH**	ICD: K758 and K701	
**Non-alcoholic fatty liver disease, NAFLD**	ICD: K760	
**Cancer**		
Gastric cardia cancer	ICD: C16	
Liver cancer	ICD: C22	
Pancreas cancer	ICD: C25	
Corpus uteri cancer	ICD: C54	
Ovary cancer	ICD: C56	
Kidney cancer	ICD: C64	
Colorectal cancer	ICD: C18	
Breast cancer	ICD: C50	
Endometrial cancer	ICD: C54	

Table A3: Definition of microvascular comorbidities (only defined for patients with type II diabetes)

Comorbidity	ICD-10 diagnosis code	
**Diabetes Type II**		**See appendix 2*
Diabetic mono-/polyneurotherapy	ICD:	G99.0, G59.0, G63.2, E11.4, E12.4, E13.4, E14.4
Diabetic eye complication	ICD:	H28.0, H35.8, H36.0, E11.3, E12.3, E13.3, E14.3
Diabetic foot complication	ICD:	E11.68, M14.2, M14.6, M90.8, L98.4, E11.5, E12.5, E13.5, E14.5
Diabetic kidney disease	ICD:	N08.3, E11.2, E12.2, E13.2, E14.2

Appendix 2: Definition of comorbidities based on guidelines from The Danish Health Data Authority

Asthma

Persons with minimum one purchase of prescription medication with a labelled indication for asthma (indication codes 202, 203, 822 in the Danish National Prescription Registry).

Persons who have had a at least one purchase of montelukast (ATC code: R03DC03), which is only indicated for treatment of asthma.

Persons with minimum one asthma-related hospital contact, i.e. a hospital contact where the ICD code J45 and sublevels has been listed as primary or secondary diagnosis.

Dementia

Persons who have had a at least one purchase of prescription medicines indicated for treatment of dementia (ATC code: N06D including sublevels).

Persons with minimum one hospital contact related to dementia, i.e. a hospital contact where the ICD code F00 – F03 including sublevels has been listed as primary or secondary diagnosis.

Type 1 diabetes

Persons who have had a at least one purchase of prescription medicines indicated for treatment of type 1 diabetes (ATC code: A10A including sublevels excluding A10AE54 and A10AE56).

Persons with minimum one hospital contact related to type 1 diabetes, i.e. a hospital contact where the ICD code E10 including sublevels has been listed as primary or secondary diagnosis.

Exclusion criteria:

Persons with type 2 diabetes.

Type 2 diabetes

Persons who have had a at least one purchase of prescription medicines indicated for treatment of type 2 diabetes (ATC code: A10AE54, A10AE56 and A10B including sublevels excluding A10BJ02).

Persons with minimum one hospital contact related to type 1 diabetes, i.e. a hospital contact where the ICD code E11 including sublevels has been listed as primary or secondary diagnosis.
